# Supplementary material for: Herpesvirus Entry Mediator as an Immune Checkpoint Target and a Potential Prognostic Biomarker in Myeloid and Lymphoid Leukemia
Source: Biomolecules. 2024 Apr 27;14(5):523. doi: 10.3390/biom14050523 (PMC11117912; doi:10.3390/biom14050523)
Supplement: Supplementary file 1 [file biomolecules-14-00523-s001.zip › biomolecules-2939267-supplementary.pdf]

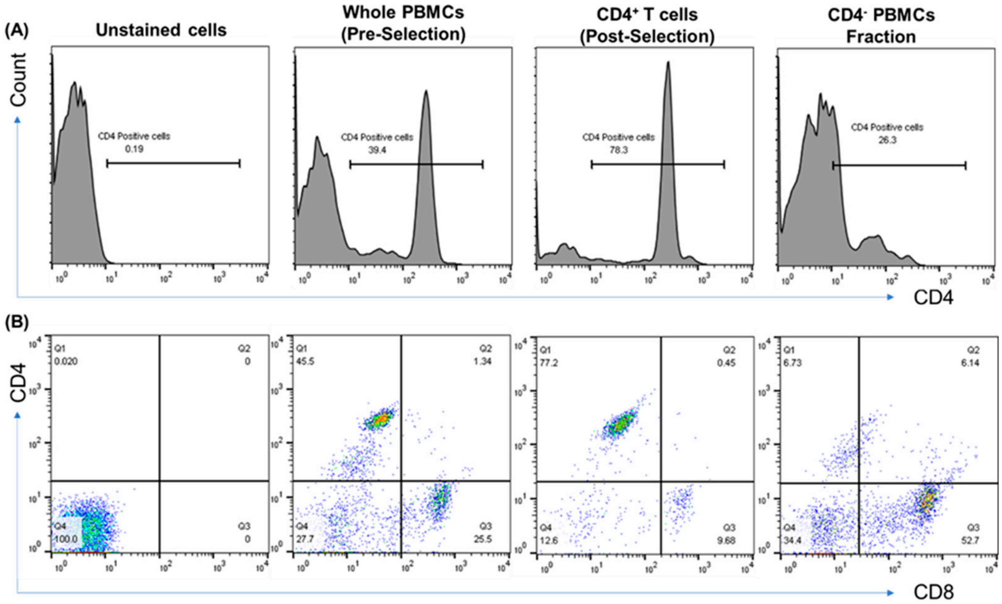

Supplementary Figure S1. Flow cytometric analysis of CD4+ T cell purification. (**A**) Histogram graphs representing the counts cells and CD4 expression throughout the different separation steps (Top: 1st unstained, 2nd pre-selection, 3rd post-selection, 4th CD4- fraction). (**B**) Dot plot graphs of cells stained with anti-CD4 (vertical axis) and anti-CD8 mAbs (horizontal axis) (Bottom: 1st unstained, 2nd pre-selection, 3rd post-selection, 4th CD4- fraction). Results are collected from 3 separate experiments.
